# Can the Brain’s Thermostatic Mechanism Generate Sleep-Wake and NREM-REM Sleep Cycles? A Nested Doll Model of Sleep-Regulating Processes

**DOI:** 10.3390/clockssleep6010008

**Published:** 2024-02-19

**Authors:** Arcady A. Putilov

**Affiliations:** Laboratory of Sleep/Wake Neurobiology, Institute of Higher Nervous Activity and Neurophysiology of the Russian Academy of Sciences, 117865 Moscow, Russia; putilov@ngs.ru

**Keywords:** REM sleep, sleep cycle, two-process model, sleep-wake regulation

## Abstract

Evidence is gradually accumulating in support of the hypothesis that a process of thermostatic brain cooling and warming underlies sleep cycles, i.e., the alternations between non-rapid-eye-movement and rapid-eye-movement sleep throughout the sleep phase of the sleep-wake cycle. A mathematical thermostat model predicts an exponential shape of fluctuations in temperature above and below the desired temperature setpoint. If the thermostatic process underlies sleep cycles, can this model explain the mechanisms governing the sleep cyclicities in humans? The proposed nested doll model incorporates Process s generating sleep cycles into Process S generating sleep-wake cycles of the two-process model of sleep-wake regulation. Process s produces ultradian fluctuations around the setpoint, while Process S turns this setpoint up and down in accord with the durations of the preceding wake phase and the following sleep phase of the sleep-wake cycle, respectively. Predictions of the model were obtained in an *in silico* study and confirmed by simulations of oscillations of spectral electroencephalographic indexes of sleep regulation obtained from night sleep and multiple napping attempts. Only simple—inverse exponential and exponential—functions from the thermostatic model were used for predictions and simulations of rather complex and varying shapes of sleep cycles during an all-night sleep episode. To further test the proposed model, experiments on mammal species with monophasic sleep are required. If supported, this model can provide a valuable framework for understanding the involvement of sleep-wake regulatory processes in the mechanism of thermostatic brain cooling/warming.

## 1. Introduction

The basic properties of biological time-measuring systems have easily lent themselves to mathematical modeling. For the last four decades, a quantitative version of the two-process model of sleep-wake regulation [1] remains the major source of our understanding of the basic mechanisms generating the sleep-wake cycle. The authors of a pioneering publication noted a close resemblance between a thermostat and Process S, i.e., the hypothetical process of homeostatic regulation of sleep duration and intensity proposed by their model [1]. Later, the analogy between this somnostat [1] and a thermostat was further supported by demonstrating that a model of a relay thermostat [2] could serve as a technical counterpart to the model of Process S [3,4].

An all-night sleep episode in humans is separated into several approximately 90 min sleep cycles. They are usually yielded by an analysis of polysomnographic records as the alternations between two sleep substates, NREM (non-rapid-eye-movement) sleep and REM (rapid-eye-movement) sleep. Such sleep cycles are found only in two groups of terrestrial vertebrates, mammals and birds. Although these groups evolved independently from one another, they developed a common adaptation, thermal homeostasis (i.e., they became homeothermic). Therefore, it comes as no surprise that several research groups hypothesized that a thermostatic brain cooling/warming process underlies these alternations between NREM and REM sleep [5,6,7,8,9,10,11,12]. In particular, animal studies provided evidence for an elevation in brainstem temperature during wakefulness. If wakefulness can impose “thermal load” on the brain, it can be dissipated during the following NREM sleep [5]. A decrease in brainstem temperature during NREM sleep was found to be associated with a reduction in both neuronal metabolism and energy consumption. In contrast, brainstem temperature rises during the following REM sleep, and this rise is associated with increased neuronal metabolism [12,13]. Consequently, the brainstem thermoregulation hypothesis of the NREM-REM sleep cycle postulates that the REM sleep phase is triggered by a decrease in brainstem temperature during the preceding NREM sleep phase. Such a temperature-regulating mechanism prevents further warming-up by the termination of the REM sleep phase after recognizing the return to the target temperature [12].

If a thermostat is designed to regulate temperature, can a model of this device be applied to modeling two hypothetical sleep regulatory processes generating the circadian sleep-wake cycles and ultradian (NREM-REM) sleep cycles? The major aim of the present *in silico* and simulation studies was to try to answer this question and determine whether an additional, model-based argument can be provided in support of the hypothesis that the thermostatic brain cooling/warming process can underlie the observed sleep cyclicities. Consequently, the major tested hypothesis was the expectation of a resemblance between the shape of fluctuations in temperature in a thermostat model and the shape of fluctuations in spectral electroencephalographic (EEG) indexes of sleep regulation in human sleep. Such applicability of a simple model of a thermostatic regulator for the prediction and simulation of the circadian and ultradian cyclicities in humans can further support the hypothesis that REM sleep reverses the reductions in brain temperature and metabolism during preceding NREM sleep. Moreover, the present results can help to suggest which animal experiments are required for future examination of this hypothesis.

## 2. Results

It was previously proposed [3,4] that a model of a relay thermostat can serve as a technical counterpart of Process S or, in other terms, a homeostatic process or somnostat [1]. Here, the classical two-process model (Formula (3) [1,14] was extended by including Process s generating sleep cycles (Formula (4)) into Process S generating human sleep-wake cycles (Formulas (3) and (5)). In the present *in silico* study (Section 2.1), the amplitude of and variation in the shape of fluctuations of the sleep-regulating process above and below its desired setpoint were predicted and these predictions were tested by simulation of the oscillations of spectral EEG markers of the sleep-wake regulatory processes *S*(*t*) and *s*(*t*) obtained in human sleep studies (Section 2.2 and, additionally, Section S2.6 of Supplementary Material).

### 2.1. Results of In Silico Study

**Figure 1:**
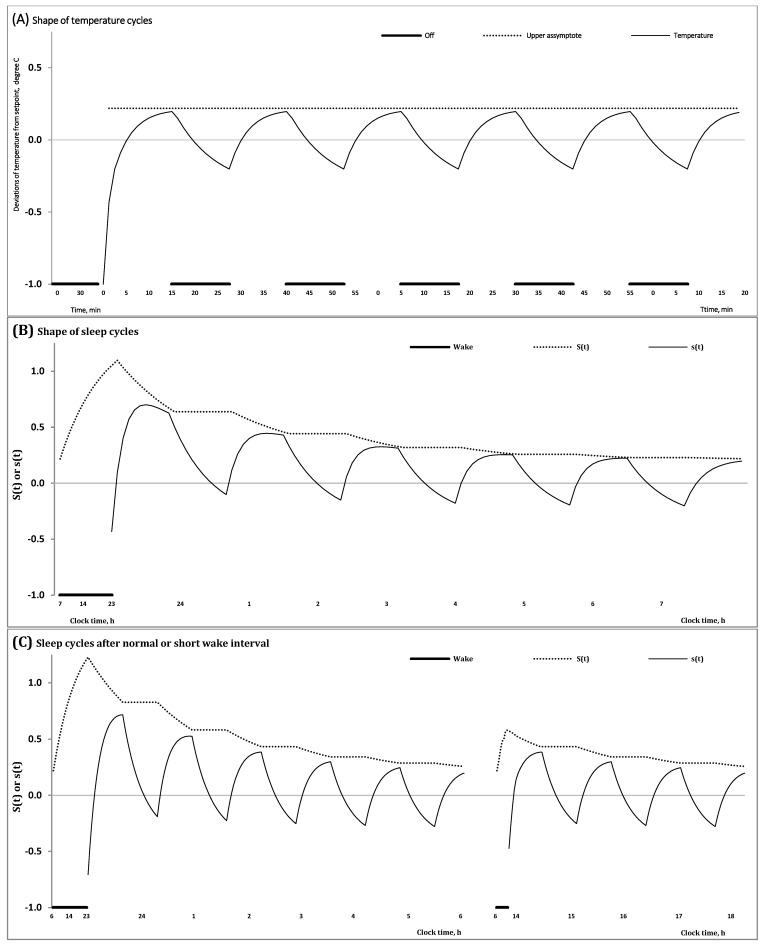
Results of *in silico* study of temperature and sleep cycles. (**A**–**C**) In the present *in silico* study, temperature and sleep cycles were computed to predict the shape of fluctuations in temperature and the variation in the mean level, amplitude and shape of NREM-REM sleep cycles during a sleep episode, i.e., the dependence of these parameters of the oscillating process on the duration of the preceding wake phase of the sleep-wake cycle. (**A**) Dynamics of temperature and the upper asymptote (maximal temperature) in the model of the relay thermostat: In this simple model, the maximal temperature does not change from one temperature cycle to another. See Equations (S5) and (S6) in Supplementary Material. (**B**,**C**) Dynamics of the two processes of regulation of the sleep cyclicities, *S*(*t*) or *s_u_*(*t*) and *s*(*t*), predicted by the model combining ultradian (internal) and circadian (external) somnostats: Dynamics during two (wake and sleep) phases of the sleep-wake cycle and during either a normal or a shorter sleep episode after either a normal or a shorter duration of the wake phase of the sleep-wake cycle are shown in (**B**) and ((**C**), left) or ((**C**), right), respectively. Unlike the upper asymptote in the thermostat model, (**A**), the upper asymptote in the proposed model, *S*(*t*) *= s_u_*(*t*) (Formula (3) = (5)), varies in accord with the exponential low, (**B**,**C**). As a result, such characteristics of the NREM-REM sleep cycle as its mean level, amplitude and shape vary throughout the sleep episode. See Equations (3)–(5) in Section 4.

A relay thermostat is designed to regulate temperature [2]. Its aim is to perform actions directed at maintaining a desired setpoint. By seeking to reduce the error between desired and measured temperatures, the thermostat exerts control by switching a heating/cooling device on or off. However, such heating/cooling for the purpose of maintaining a desired setpoint temperature inevitably produces hysteresis which is defined as the expected deviation of temperature from its target setpoint. Therefore, before the heating/cooling is triggered, this deviation allows for a certain amount of temperature fluctuation above and below the desired temperature setpoint [2]. As the lowest/highest temperature that was allowed below/above the temperature setpoint is reached, heating/cooling is switched to bring temperature closer to the desired setpoint temperature (Figure 1A).

Equations (S5) and (S6) in Supplementary Material suggest that, in the model of a relay thermostat, each temperature cycle can be mathematically described as an alternation of an inversed exponential buildup with a compensating exponential decay [2,3,4]. Formally, the classical version of the two-process model of human sleep-wake regulation [1] proposes that identical oscillations of the regulated parameter can be used for the mathematical description of the approximately 24 h alternations of wake and sleep phases of the sleep-wake cycle governed by the homeostatic Process S (Formula (3)) [3,4]. Namely, it was hypothesized by the authors of the classical model [1,14] that the shape of fluctuations of Process S, a regulator of the human sleep-wake cycle within an approximately 24 h period, consists of two—wake and sleep —phases that can be described using simple—inverse exponential and exponential—functions (Formulas (3a) and (3b), respectively). Later, the circadian term (Formula (2)) was added in (Formula (3)) to incorporate the modulation of all parameters of Process S by a sine function with circadian period representing the mechanism of control of the circadian clocks over the sleep-wake cycle (Formula (1)) [3].

In the proposed model (Formulas (1)–(5)) (Table 1), one more process, Process s, a governor of NREM-REM sleep cycles (Formula (4)), was additionally incorporated into Process S, a governor of sleep-wake cycles (Formulas (3) and (5)). Each NREM-REM sleep cycle consists of two—the fist and the second—phases. The first phase is dominated by NREM sleep, while the second phase includes REM sleep. Even after including Process s into the model, exactly the same shape of fluctuations in the regulated parameter can be proposed for modeling the cyclicities with different periods generated by processes S and s (Table 1). For instance, the ultradian (NREM-REM) sleep cycles controlled by Process s can be modeled as an inverse exponential buildup during the first phase and a compensating exponential decay during the second phase (Formula (4)), i.e., as it is proposed for the classical version of Process S [1] (Figure 1B).

Consequently, the proposed model includes, in total, three major regulating processes (Formulas (1)–(5)) with the most important characteristics listed in Table 1. The model posits that the oscillations produced by the classical Process S (or circadian somnostat or sleep-wake homeostatic process) within an approximately 24 h period [1] reflect the process of tuning the setpoint of another homeostatic process (or Process s or ultradian somnostat) producing NREM-REM sleep cycles. The duration of the preceding wake phase determines this setpoint in the beginning of the following sleep phase of the sleep-wake cycle. After a wake episode of longer duration, this setpoint is elevated at a higher level than that after an episode of shorter duration (Figure 1C). During sleep cycles, Process S tunes this setpoint in accord with the exponential law on the interval of the first phases of each sleep cycle (Figure 1B,C). The model can be named a “nested doll model” because one of the regulatory mechanisms (Process s) produces sleep cycles with a short (ultradian) period that are “nested” in one of two phases of the human sleep-wake cycle with a long (circadian) period produced by another regulatory mechanism (Process S). If Process s is aimed at maintaining the desired setpoint, Process S is aimed at determining which of the possible setpoints is desired after a preceding wake phase of certain duration of the sleep-wake cycle and how much this setpoint can be lowered during each of the following sleep cycles of the sleep phase of the sleep-wake cycle (Table 1). Consequently, unlike the setpoint in the model of the thermostat (Figure 1A), the setpoint of Process s varies (Figure 1B,C). It changes throughout the preceding wake phase of the sleep-wake cycle and during the first phase of each of the following NREM-REM sleep cycles. In other words, Process S is designed to regulate the rise of setpoints during the preceding wake phase of the sleep-wake cycle (Figure 1C) and its reduction from one cycle to another during the following sleep phase of the cycle (Figure 1B,C).

The model predicts the change in such characteristics of the NREM-REM sleep cycle as its mean level, amplitude and shape throughout a sleep episode. For instance, the amplitude of the cycle declines during such an episode (Figure 1B). Moreover, the amplitude of the first cycles varies in accord with the duration of preceding wake phases because the extent of elevation of the setpoint prior to sleep onset depends upon this duration. Namely, the amplitude of oscillations produced by Process s must be larger after a longer wake phase than that after a shorter wake phase (Figure 1C).

If the shape of fluctuations in temperature in the thermostat does not change from one cycle to another (Figure 1A), the proposed model of sleep regulation predicts that the shape of fluctuations of Process s varies from one cycle to another due to the exponential decay of the setpoint controlled by Process S (Figure 1B). Following Achermann and others [15,16], such rather complex shapes of the first cycles of an all-night sleep episode can be separated into rising, saturation and decaying sections. It was previously proposed that simulation of such complex shapes requires a quite long formula compared to the simple mathematical formulation of the classical homeostatic process [15,16]. Notably, although the model-predicted shape of the first sleep cycles of an all-night sleep episode is rather complex and varies from one cycle to another, this complexity and variation are explained by the superposition of simple exponential functions (Formulas (3) and (4)) (Figure 1B,C).

If the mean level of fluctuations in temperature in the thermostat does not change from one cycle to another (Figure 1A), the sleep regulation model predicts that the mean level of Process s declines throughout the sleep episode due to the exponential decay of the setpoint, controlled by Process S. Notably, the interval with negative (below zero) values gradually increases in a sequence of NREM-REM sleep cycles (Figure 1B,C).

Therefore, the following model-based predictions were obtained in the *in silico* study:

− The duration of the previous wake phase determines the mean level, amplitude and shape of fluctuations of the regulated process on the interval of the first sleep cycles constituting the sleep phase of the sleep-wake cycle;
− The shape of these cycles cannot closely resemble the alternations of simple exponential curves;
− The interval with negative values of the regulated process gradually increases in a sequence of sleep cycles during an all-night sleep episode.

### 2.2. Results of Simulation Study

Table 2 lists the model’s parameters used in the simulation study. These parameters were obtained by fitting the time courses of spectral EEG indicators of processes S and s, *S*(*t*) and *s*(*t*).

**Figure 2:**
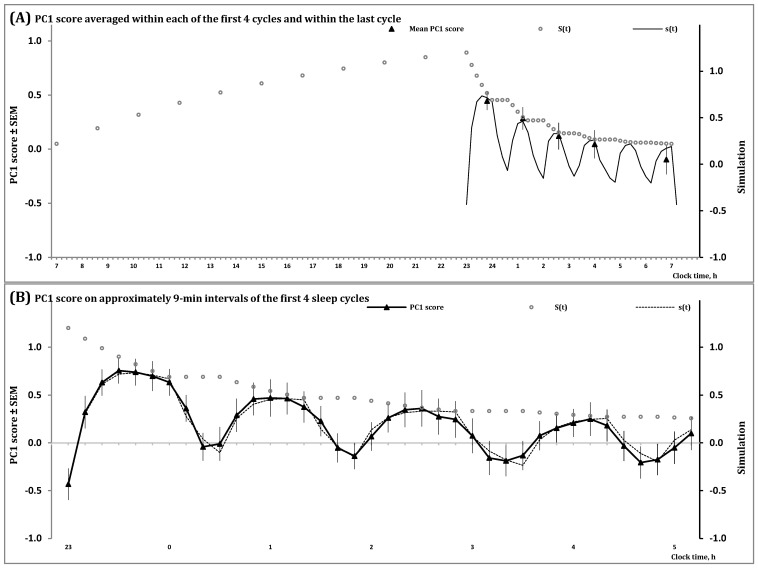
Simulation of data on sleep regulation during all-night sleep.

In Figure 2 and Figure S1, the processes regulating the wake and sleep phases of the 24 h sleep-wake cycle, *S*(*t*), and the first and second (NREM and REM) phases of sleep cycles, *s*(*t*), are shown during an all-night sleep episode. They illustrate that the predictions of the *in silico* study were confirmed by simulation of data on a spectral EEG indicator of processes S and s in an all-night sleep episode. This study predicted that the amplitude and shape of the first sleep cycles vary due to the influence of the exponential decay of Process S, the regulator of the sleep-wake cycle, on the inverse exponential buildup of Process s, the regulator of sleep cycles (Figure 1B,C). Notably, the shapes produced by each of these two processes, S and s (Formulas (3)–(5)), are formally identical to the simple shape of cycles of temperature in a thermostat [2], i.e., Formulas (S5) and (S6) in Supplementary Material. In the case of the process regulating sleep cycles (Process s), the model suggested a mathematically simple shape of the cycle consisting of an inverse exponential buildup of the regulated parameter during the first (NREM) phase of each cycle with a compensating exponential decay of the regulated parameter during the second (REM) phase of the cycle. However, such a simple shape can be observed only near the end of the all-night sleep episode when the decay of Process S becomes very slow (Figure 1B,C) and very close to the baseline upper asymptote of the model (Figure 1). In contrast, the first cycles of this all-night sleep episode have rather complex shapes (Figure 1B,C) and they were separated into rising, saturation and decaying sections in the previous simulations of sleep cycles [15,16]. The presented model predicted (Figure 1B) and the simulations of data confirmed (Figure 2) that none of the shapes of the first three sleep cycles resemble a mathematically simple shape consisting of the alternation of an inverse exponential buildup in the first phase of each sleep cycle with an exponential decay in its second phase. Instead, the influence of Process S on a setpoint of Process s causes the deviation from a simple inverse exponential buildup described in [15,16] as two, rising and saturation, sections of the three-section shapes of the oscillations of a marker of sleep regulation on the interval of the first phase of a sleep cycle. Unlike this first phase, the following second phase can be represented by a simple exponential form [15,16].

In the present simulation study, simulations of sleep cycles in an all-night sleep episode were performed on an example of one of the spectral EEG markers of sleep regulation, PC1 score. The processes of sleep-wake regulation, *S*(*t*) and *s*(*t*), are illustrated on the intervals of (A) two (wake and sleep) phases of the human sleep-wake cycle and (B) the first four sleep cycles of an all-night sleep episode. On each of these intervals of inverse exponential buildup of PC1 score during the first phase of sleep cycles (Formula (4a)), Process S governs an exponential decay of the setpoint of Process s (Formula (5a)). Therefore, a rather complex shape of the first sleep cycles can be explained by the modulating influence of a mathematically simple exponential decay of the setpoint (Formula (5a)) on a similarly simple inverse exponential buildup of PC1 score during the first phase of these cycles (Formula (4a)). See also Section 4 for the model’s parameters and Supplementary Material for the details on estimation of PC1 score.

**Figure 3:**
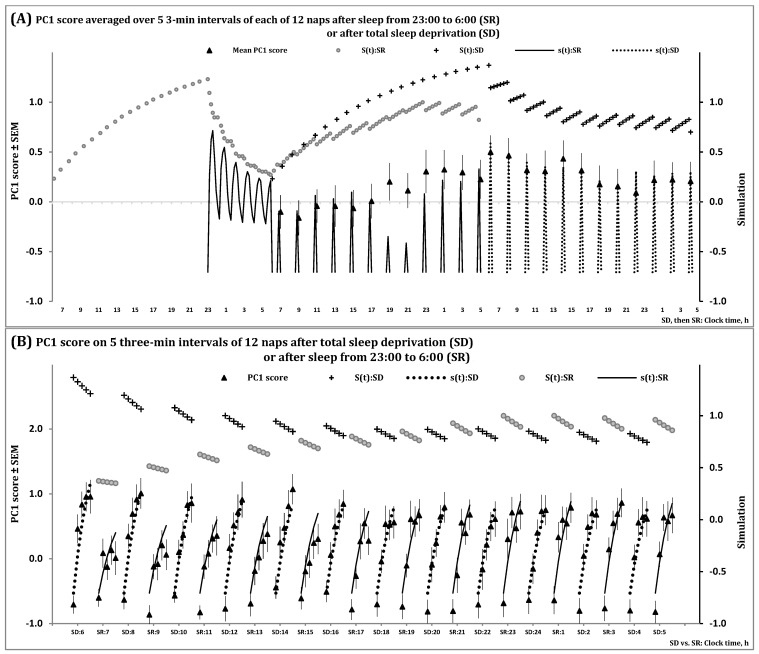
Simulation of data on sleep regulation in 12 naps.

Figure 3 and Figure S2 illustrate the spectral EEG indicators of the processes regulating the wake and sleep phases of the 24 h sleep-wake cycle, *S*(*t*), and the first and second (NREM and REM) phases of sleep cycles, *s*(*t*), during 12 naps following sleep deprivation and sleep restriction. Particularly, Figure 3 shows that the predictions of the *in silico* study were confirmed by data on a spectral EEG indicator of processes S and s obtained for short sleep episodes observed in multiple naps. The model predicted (Figure 1C) that the variation in the level of the setpoint of Process s explains the observed variation in steepness of the buildup of the spectral EEG indicator in the beginning of each napping attempt. The longer the intervals of the preceding wake phase(s), the steeper the buildups, because a longer interval of wakefulness results in a higher buildup of the setpoint governed by Process S (Figure 3A,B). When one group of study participants were sleep-deprived while another group slept for 7 h prior to 12 20 min napping attempts, these buildups were profoundly steeper in the former than in the latter group in the first napping attempts. However, the difference in the steepness of buildups between the groups gradually disappeared because study participants were not allowed to sleep longer than 20 min during the following day and night (Figure 3B).

In the present simulation study, simulations of sleep during 12 napping attempts were performed. (A) The time courses of the processes of sleep-wake regulation, *S*(*t*) and *s*(*t*), during two days: *S*(*t*) is shown on the left side of the graphs for two (wake and sleep) phases of the process of sleep-wake regulation under conditions of sleep restriction from 23:00 to 6:00 (SR group) and it is shown in the central part of the graphs for the condition of total sleep deprivation (SD group). The following 12 20-min napping attempts across the 24 h time interval are shown in the central part for SR and on the right side for SD. (B) Simulation of the same processes *S*(*t*) and *s*(*t*) during these napping attempts are shown in more details, across the intervals of the first 12 min of a sleep episode in each of the 12 napping attempts, after either total sleep deprivation (12 naps at every even hour starting at 6:00) or restriction of sleep to 7 h (12 naps at every odd hour starting at 7:00). Process S regulates both the length and intensity of the sleep phase of the sleep-wake cycle in accord with duration of the preceding wake phase. In terms of the model, Process S (Formula (5)) tunes the setpoint of Process s (Formula (4)) by elevating it during the wake phase and reducing it during the first phase of each of the following sleep cycles. Since the SD group did not sleep while the SR group slept for 7 h prior to napping attempts, the buildup of PC1 score after initiation of sleep was profoundly steeper in the former group compared to the latter group (left part of the graphs). The difference between the groups gradually disappeared because a nap longer than 20 min was not allowed during the following day and night hours (right part of the graphs). The curves of *S*(*t*):SD and *S*(*t*):SR across the intervals of each nap illustrate the effect of preceding wakefulness on the level of the setpoint determining the steepness of buildup. See also Section 4 for the model’s parameters and Supplementary Material for details on the estimation of PC1 score.

Thus, the model provides a possibility to solve the problem of parsimony of mathematical descriptions of the time courses of spectral EEG markers of sleep regulation in a sequence of sleep cycles of an all-night sleep episode. It predicts such complexity of shape despite the mathematically simple (exponential) form of the phases of oscillations generated by processes S and s. The influence of one process on the other produces the observed rising and saturation sections of the cycle across the interval of its first phase (Formula (5a)), whereas, due to the absence of such influence across the interval of the second phase of sleep cycle (Formula (5b)), it is described as a simple exponentially decaying section of the cycle (Figure 2B).

The model also predicts such a well-known fact as a gradual increase in the interval of REM sleep from one NREM-REM sleep cycle to another throughout an all-night sleep episode. Given that stages N2 and N3 of NREM sleep are characterized by positive PC1 scores while PC1 score is negative for other stages [17], the increase in the interval of negative PC1 scores in a sequence of sleep cycles reflects a gradual increase in duration of REM sleep during the end of cycle (Figure 2B) that was predicted in the *in silico* study (Figure 1B,C).

## 3. Discussion

The proposed nested doll model postulates oscillations of the regulated parameter with two distinct periods produced by two homeostatic processes, S and s. Process s produces ultradian (NREM-REM) sleep cycles that constitute the sleep phase of the circadian sleep-wake cycle produced by Process S. The purpose of Process s is to control the ultradian oscillations above and below a desired setpoint, while Process S determines which of the possible levels of the setpoint are desired in the current moment. Due to such “regulating the regulator” effect, the level of the setpoint at which sleep is initiated depends upon the duration of the previous wake phase, and this level decays during the following sleep phase of the sleep-wake cycle until it reaches its baseline level at the end of this phase. As predicted by the model of the thermostat, only simple exponential functions are required for prediction and simulation of the oscillations of spectral EEG indicators of sleep regulation in a sequence of sleep cycles. Notably, the nested doll model predicts that the first sleep cycles across the interval of an all-night sleep episode have rather complex and varying shapes instead of resembling alternations between inverse exponential buildups and compensating exponential decays. The model explains these shapes by the influence of an exponential decay of the setpoint governed by Process S during an inverse exponential buildup of the regulated parameter governed by Process s. Despite the rather complex shapes of these oscillations in the first sleep cycles of the all-night sleep episode, they were reproduced by simulating the oscillations of spectral EEG indicators of sleep regulation. Since Process S also tunes the setpoint across the interval of the preceding wake phase of the sleep-wake cycle in accord with the duration of this phase, its influence explains the observed variation in the steepness of the buildup governed by Process s in the beginning of the sleep cycle. A buildup was predicted to be steeper after a longer interval of wakefulness, and this relationship was reproduced in the simulations of this buildup in multiple napping attempts after different intervals of preceding wakefulness. Therefore, the present model-based results supported the assumption of identity of the mathematical descriptions of the mechanisms underlying NREM-REM sleep cyclicities and fluctuations in temperature above and below the desired setpoint.

### 3.1. A Model-Based Support for the Hypothesis of Brainstem Thermoregulation Function

These results are consistent with the hypothesis of the brainstem thermoregulating function of the processes underlying sleep cycles [12]. In the framework of this hypothesis, a regulatory role of Process s can be interpreted as the following: The time course of spectral EEG indexes of sleep regulation reflects the work of the brain’s thermostat that heats or cools to a setpoint brain temperature. By seeking to reduce the error between the setpoint and current temperature, it produces hysteresis which explains the sleep cycle consisting of two—the first (NREM sleep) and the second (REM sleep)—phases. Since brain heating occurs during the wake phase of the sleep-wake cycle, the process of cooling (i.e., NREM sleep) is always triggered after sleep onset, when the temperature deviates above this setpoint during preceding wakefulness. During the phase of NREM sleep initiated after termination of the wake phase, temperature crosses the setpoint and continues its exponential decay until triggering the process of heating around the transition from NREM sleep to REM sleep. During the phase of REM sleep, temperature crosses the setpoint in the opposite direction and continues its exponential buildup until triggering the process of cooling during the transition from REM sleep back to NREM sleep.

In the framework of the hypothesis of brain thermostatic cooling/warming, the regulatory role of Process S can be interpreted as the following: Since brain heating occurs during wakefulness, a longer interval of wakefulness is associated with a longer period of brain heating, whereas a shorter interval is associated with a shorter period of brain heating. Therefore, a setpoint of brain temperature requires correction to a higher or lower level depending upon the duration of the previous wake phase. If a setpoint is tuned to a higher level due to further heating during prolongation of wakefulness, the cooling process initiated by sleep onset is accelerated. Notably, the model predicts that such overheating occurs even during a normal human wake phase of the 24 h sleep-wake cycle. Given that our species evolved to have monophasic sleep with only one wake phase occupying two-thirds of the length of the 24 h sleep-wake cycle, such correction reveals itself in the baseline time course of a spectral EEG indicator of the sleep-regulating Process S described in [1,14]. Therefore, the model proposes that Process S can be regarded as the regulator controlling the return of the setpoint to its baseline level in the last sleep cycle of an all-night sleep episode. The model predicts that the setpoint is exponentially decaying during each of the NREM sleep phases of, at least, the first three sleep cycles. Due to such exponential decay produced by Process S, the buildup of the spectral EEG indicator during each of the first phases of these cycles produced by Process s deviates from its simple exponential form. Therefore, such deviation can be revealed in the form of two—exponential and saturation—sections of the three-section shape of the sleep cycle [15,16] (unlike this first phase of the cycle, the following second phase can be modeled with a simple exponential function [15,16] because the decay of the setpoint occurs only during the first phase).

Overall, the present results provided a model-based argument in support of the hypothesis linking sleep cyclicities (i.e., the sleep-wake cycle and NREM-REM cycles) to the brain’s thermostatic cooling/warming mechanism. The brainstem thermoregulation hypothesis [12] considers a decrease in brainstem temperature during the NREM phase as the trigger of initiation of the following REM phase. The NREM-REM cycle repeats after the termination of further warming-up, and this termination can be viewed as a response of the temperature-regulating mechanism to the return to the target brainstem temperature in the end of the REM phase [12]. A model-predicted decay of Process S during sleep can be explained by the reduction in the setpoint across sleep cycles that is in agreement with the assumption that high brain temperature during wakefulness imposes “thermal load” on the brain which is dissipated during NREM sleep [5]. Moreover, the assumption of elevation in the setpoint in response to sleep deprivation agrees with findings of animal experiments that demonstrated greater brain cooling during longer or deeper bouts of NREM sleep as part of recovery sleep following sleep deprivation [7].

### 3.2. Support from the Studies of Polyphasic Sleep in Laboratory Rodents and Infants

Given that Process S is designed to respond to the prolongation of the wake phase of the sleep-wake cycle by elevation in the level of the setpoint of Process s, it can be difficult to demonstrate such a mechanism of tuning the setpoint in species with polyphasic sleep. The contribution of Process S is expected to be small in these species due to short intervals of wakefulness prior to sleep. Fortunately, this provides a possibility to apply simple exponential functions for descriptions of Process s, i.e., an inverse exponential buildup of a regulated parameter during REM sleep and wakefulness and an exponential decay during NREM sleep. Indeed, the applicability of simple exponential functions for simulating the time courses of indicators of sleep- and temperature-regulating processes was supported by the approach previously applied by Franken et al. [18] and Sela et al. [19] for the simulation of the time courses of cortical temperature in a sequence of vigilance states in rats. They showed that an increase in temperature during wakefulness and REM sleep can be simulated by an inverse exponential function, while its decrease during NREM sleep can be simulated by an exponential function.

In addition, the results of these studies [18,19] were in line with the results of present preliminary simulations (see Supplementary Material) indicating a relatively small influence of the circadian clocks on the time course of spectral EEG indexes of sleep regulation across the interval of sleep cycles as compared to the influence of each of the three alertness states (wake, NREM and REM sleep).

The temporal structure of human sleep changes across development as it consolidates from the polyphasic sleep of infants to the single night-time sleep episode in adults [20]. It is well documented that, in infants, several alternations between the wake and sleep phases occur during a 24 h interval. The results of present simulations are also in line with data on the development of spectral EEG indicators of sleep regulation in such polyphasic sleep of infants. Since the last interval of wakefulness prior to the episode of night-time sleep is relatively short, it is expected that the effect of Process S on the setpoint of Process s in infants might be much smaller compared to the effect observed across the interval of an adult’s all-night sleep episode. Indeed, an exponential decay of spectral powers from one sleep cycle to another was detected in none of the spectral frequency ranges in the records of night-time sleep of infants that were two weeks of age [21]. As for infants of older ages, the authors of the same study [21] associated the development of the process of homeostatic sleep regulation with elevations in theta activity (5–9 Hz) in the beginning of night sleep episodes. The level of this activity was higher only in the first sleep cycle at the age of two months, whereas, at the age of nine months, it was higher already in the first three cycles [21], thus resembling the pattern of elevation in slow-wave activity (SWA) in an adult’s all-night sleep episode [1,14].

The age-associated process of consolidation of human sleep into fewer sleep episodes has been recently modelled in [20]. This process was shown to be linked to slower accumulation of sleep need in older age leading to a change in the rates of growth and decay of homeostatic sleep need that, in turn, results in a transition between polyphasic and monophasic sleep [20]. Notably, model-based simulations of age-associated effects on sleep times suggested that the difference in sleep timing between late adolescence and younger ages can be also explained by slower accumulation of sleep need in older individuals [4].

### 3.3. A Need for Further Support from Studies on Mammal Species with Monophasic Sleep

The proposed model of regulation of sleep cyclicities can provide a valuable framework for studies aimed at deepening our understanding of the mechanisms of sleep regulation and thermostatic brain cooling/warming. For instance, the presented model explains the complexity of sleep regulation in humans by the need for not only the maintenance of the setpoint by Process s but also for its turning by Process S. It seems that, in our species, such complexity evolved for the adjustment of the work of sleep regulatory mechanisms to an unusually long length of the wake phase of the sleep-wake cycle, i.e., approximately two-thirds of the 24 h period. Consequently, further support for the brainstem thermoregulation hypothesis of sleep cyclicities can be provided by the results of experiments designed to simultaneously measure the time courses of brain temperature and spectral EEG indicators of sleep regulation in a species with a similarly long duration of the wake phase of the sleep-wake cycle. Humans cannot participate in such experiments, but, fortunately, there is a small group of mammalian species, including several species of small primates, that, like our species, evolved to have monophasic sleep, i.e., only one wake phase and only one sleep phase occurring within the 24 h time interval of the cycle. The present results predict that the same Equations (3)–(5) can be used for simulation of data on both the time course of brain temperature and the time course of a spectral EEG indicator of sleep regulation from experiments on these few species. Particularly, both time courses of this temperature and this indicator are expected to reveal a strong effect of Process S on the shape of fluctuations produced by Process s.

In its present form, the model does not account for the anatomical and physiological substrate of processes S and s, i.e., the neurotransmitter-mediated interactions between neuronal populations promoting the three vigilance states, wake, NREM and REM sleep. Therefore, further development of the model in this direction is required in addition to its support by results of experimental studies of mammal species with monophasic sleep.

Moreover, it is necessary to examine the replicability of the results of the presented simulations by simulating data from larger independent samples collected in experimental studies aimed toward manipulations of the duration and timing of the sleep and wake phases of the human sleep-wake cycle.

## 4. Materials and Methods

In the theoretic framework of the classical version of the two-process model of sleep regulation [1,14], the sleep-wake cycle is generated by the hypothetical Process S or somnostat. As mentioned in the Introduction, the term “somnostat” has been used by the authors to describe quantitative versions of this model [1] to emphasize that their hypothetical homeostatic process resembles a thermostatic process. They noted that both processes produce hysteresis, i.e., a fluctuation above and below a target setpoint. Later, a relay thermostat was proposed to represent a technical counterpart of this hypothetical homeostatic process [3]. A model of a relay thermostat [2] (Figure 1A) was shown to be formally identical to the model of a somnostat, i.e., the hypothetical homeostatic process or Process S in the classical version of the two-process model of regulation of the sleep-wake cycle [1]. Supplementary Material includes a description of this thermostatic model [2] that was used for the development of a deductive version of the model of sleep-wake regulation named “rhythmostat” [3]. This Supplementary Material also contains additional description of methods of estimation of the time courses of two spectral EEG indicators of sleep regulation simulated with this model.

### 4.1. The Proposed Model of the Sleep-Wake Regulatory Processes S and s

The term “rhythmostat” [3] refers to the hypothesis of the so-called rhythmostasis [22] that is the capacity of the body to keep its rhythms nearly constant, including such diurnal rhythm as the human sleep-wake cycle. It was previously shown that circadian rhythmostasis of the sleep-wake cycle can be achieved by the modulation of parameters of Process S by circadian clocks [4]. The modulation of the process *S*(*t*) was incorporated in the thermostat = somnostat model by introducing the simplest periodic (sine) function with a circadian period, *C*(*t*), in the equations describing the time course of Process S [3] (see also Supplementary Material).

When *t*_1_ and *t*_2_ are the initial times for the buildup (wake) and decay (sleep) phases of the sleep-wake cycle (e.g., sleep offset and sleep onset, respectively), the time course of the sleep-wake regulatory process, *S*(*t*), can be described as:

(1a) $$S\left(t\right)=\left[S_{u}+C\left(t\right)\right]-\left\{\left[S_{u}+C\left(t\right)\right]-S_{b}\;\right\}*e^{-\frac{t-t1}{\left[Tb\;-\;k\;*\;C\left(t\right)\right]}}$$

(1b) $$S\left(t\right)=[S_{l}+C(t)]-\{S_{d}-[S_{l}+C(t)]\}*e^{-\frac{t-t2}{\left[Td\;-\;k\;*\;C\left(t\right)\right]}}$$

where

(2) $$C(t)=A*sin(2\pi *t/\tau +\varphi_{0})$$

is a sine function with a circadian period τ, representative of the modulating influence of the circadian clocks on *S*(*t*). In the case of a habitual sleep-wake cycle with a 24 h period, the cycle has one wake phase and one sleep phase, i.e., in humans, sleep is typically monophasic.

This sleep-wake cycle differs from the NREM-REM sleep cycle in time scale. Therefore, it is expected that this circadian modulation of the regulated parameter within each of the approximately 90 min sleep cycles cannot strongly influence the time course of Process s over such a short interval. Indeed, the preliminary simulations with *A* = 0.2 and *A* = 0.0 confirmed this expectation (Table S1). Therefore, for the sake of simplicity, *A* was reduced to zero in the simulations illustrated in Figure 2, Figure 3, Figures S1 and S2. In the absence of circadian modulation, the rhythmostatic process (1,2) [3] does not differ from Process S in [1] (see also the description of *S*(*t*) in Supplementary Material):

(3a) $$S\left(t\right)=S_{u}-{(S}_{u}-S_{b})*e^{-\frac{t-t1}{Tb}}\;\;for\;wake\;phase$$

(3b) $$S\left(t\right)=S_{l}-\left(S_{d}-S_{l}\right)*e^{-\frac{t-t2}{Td\;}}\;\;for\;sleep\;phase$$

To develop a nested doll model, it is necessary to add one more homeostatic process, Process s, *s*(*t*),that represents the alternations of two, buildup and decay, phases (4a and 4b) of the regulator of NREM-REM cycles during the sleep phase of *S*(*t*) (3b), i.e., across the interval of an all-night sleep episode of the circadian sleep-wake cycle (Figure 1B). The same Formula (3) can be applied for simulation of the time course of the Process s that generates sleep cycles:

(4a) $$s\left(t\right)=s_{u}-{(s}_{u}-s_{b})*e^{-\frac{t-t1}{tb}}\;\;for\;NREM\;phase$$

(4b) $$s\left(t\right)=s_{l}-\left(s_{d}-s_{l}\right)*e^{-\frac{t-t2}{td\;}}\;for\;REM\;phase$$

The model connecting these two regulatory mechanisms, each of which can be described with the same Equations (3) or (4), can be named a “nested doll model”. It postulates that the regulator of the long (sleep-wake) cycles with the circadian period, *S*(*t*), is formally identical to the regulator of the short (sleep) cycles with periods of approximately 90 min, *s*(*t*), and the latter regulator is “nested” in the former regulator because the sleep phase of the sleep-wake cycle is separated into several sleep cycles.

In order to accommodate Process s into the sleep phase of Process S, *S*(*t*) across the interval of sleep phase (3b) is proposed to shift a setpoint maintained by *s*(*t*). Therefore, it is postulated that, in the equations of Process s (4a) and (4b), the role of upper asymptote *s_u_*(*t*) is played by the regulator of the circadian sleep-wake cycle *S*(*t*), i.e., *s_u_*(*t*) = *S*(*t*). During the sleep phase of the 24 h sleep-wake cycle (3b), this *S*(*t*) decays across the intervals of each of the first three first (NREM) phases of sleep cycles (5a). Such exponential decay of *S*(*t*) suggests that *s_u_*(*t*) = *S*(*t*) (3b,4a) is decaying in accord with the exponential low during the first three sleep cycles. During each of the following second (REM) phases, *s_u_*(*t*) is suggested to remain at the level reached in the preceding NREM phase because *s_u_*(*t*) is not involved in calculation of *s*(*t*) during the phase (4b). This assumption based on formal features of the model agrees with the assumptions about Process S during REM sleep in previously published simulations (i.e., [15,16]).

Consequently, the upper asymptote of the decaying *s_u_*(*t*) on the interval of the first NREM phase, from *t ≥ t_b_*_1_ *≥ t*_2_ to *t ≤ t_d_*_1_, and on the following interval of the first REM phase, from *t > t_d_*_1_ to *t ≥ t_b_*_2_, can be described as:

(5a) $$s_{u}\left(t\right)=s_{u}-{(S}_{t2}-s_{u})*e^{-\frac{t-tb1}{Tb}}\;\;for\;the\;first\;(NREM)\;phase$$

(5b) $$s_{u}\left(t\right)=S\left(t_{d1}\right)\;\;for\;the\;second\;(REM)\;phase$$

where *S_t_*_2_ is an initial value of *s_u_* at the moment of transition from the wake to sleep phase of the sleep-wake cycle (*t ≥ t_b_*_1_ *≥ t*_2_) and *s_u_* is the upper asymptote for *s*(*t*) determined by *S*(*t*) (4b). This *S*(*t*) is exponentially decaying during the first (NREM) phase of the first sleep cycle (*t ≥ t_b_*_1_ *≥ t*_2_ to *t ≤ t_d_*_1_) from its initial value at *t*_2_ to a somewhat lower value at *t_d1_*. *S*(*t*) remains practically the same (i.e., equal to this somewhat lower value) during the following second (REM) phase of sleep cycle (from *t > t_d_*_1_ to *t ≥ t_b_*_2_).

This formulation can be also applied to all following sleep cycles (Figure 1B), i.e., at the time subintervals of the sleep phase of the 24 h sleep-wake cycle, from *t ≥ t_b_*_2_ to *t ≤ t_d_*_2_ and from *t > t_d_*_2_ to *t ≥ t_b_*_3_ in the second sleep cycle, from *t ≥ t_b_*_3_ to *t ≤ t_d_*_3_ and from *t > t_d_*_3_ to *t ≥ t_b_*_4_ in the third sleep cycle, from *t ≥ t_b_*_4_ to *t ≤ t_d_*_4_ and from *t > t_d_*_4_ to *t ≥ t_b_*_5_ in the fourth sleep cycle, from *t ≥ t_b_*_5_ to *t ≤ t_d_*_5_ and from *t > t_d_*_5_ to *t ≥ t_b_*_6_ in the fifth sleep cycle, etc. However, the preliminary simulations (Table S1) suggested that, as expected from data of experimental sleep studies (see the next section), *s_u_*(*t*) almost reaches its lowest level during the first (NREM) phase of the fourth sleep cycle, at *s_u_*_4_. Therefore, when *t > t_d_*_4_, the decay became very small:

(6) $$S\left(t>d4\right)\approx S\left(t_{d4}\right)\;\;for\;any\;phase$$

As a result, *S*(*t_d_*_4_) almost reaches the level of this asymptote of *s*(*t*) at which the wake phase of a new 24 h sleep-wake cycle is initiated at *t* = *t_1_* + 24 h (Figure 1B).

### 4.2. Estimation of Spectral EEG Indicators of Processes S and s

In the pioneering publication of Borbély [14] and Daan et al. [1], two phases of Process S were proposed to be simulated by mathematically very simple exponential functions. An exponential decay of SWA in a sequence of ultradian (NREM-REM) sleep cycles can serve as a spectral EEG marker of sleep regulation during the sleep phase of the cycle. This decay is preceded by a hypothetical compensating reverse exponential buildup during wakefulness [1,14].

Numerous experimental studies were encouraged by this model [1] and their results indicated that the initial value of this exponential function changes in the model-predicted manner after any manipulations with duration of the previous wake phase. For instance, an effect of prolongation of wakefulness beyond habitual bedtimes can be modeled as an elongated inverse exponential buildup of the regulated parameter. This elongation results in a longer duration and a higher intensity of the following exponential decay of the regulated parameter. Compared to the levels of SWA in baseline sleep episodes, elevated levels of SWA were found in the first three sleep cycles of recovery sleep initiated right after such prolongation of wakefulness [1,14,23,24]. In contrast, a nap occurring in the time interval preceding habitual bedtimes can be mathematically represented by a shorter inverse exponential buildup of Process S, resulting in a shorter and less intense decay of this Process S after initiation of sleep during this nap. As predicted, the level of SWA reached in the beginning of such a nap was lower than the level obtained in the beginning of baseline sleep [25].

Further, Acherman and others [15,16] proposed an extended version of the two-process model [1] by adding the ultradian process explaining an approximately 90 min oscillation of SWA during an all-night sleep episode, i.e., an alternation between phases of NREM and REM sleep in each of 4–6 sleep cycles. The abovementioned increase in SWA in the first three NREM-REM sleep cycles of a recovery sleep episode can be measured not only as an increase in the cycle-averaged levels of this activity but also as the rate of its rise in the beginning of each cycle [23,26]. Acherman and others [15,16] proposed a model for simulation of the cycling ultradian process through fitting the time course of SWA on three subintervals of each sleep cycle. These are a cycle’s rising, saturation and decaying sections.

In previously published papers (e.g., [27]), empirical results on time courses of two spectral EEG indexes, SWA and score on the first principal component (PC1) of the EEG spectrum, were reported and illustrated in figures. All details concerning the previously published methods of collection, results of analysis and illustrations of the data on these time courses are included in Supplementary Material.

Table 1 describes the major components of the model (3–5), and Figure 1 illustrates the model’s predictions obtained in the *in silico* study and confirmed in the simulation study. The model’s parameters used in this simulation study are listed in Table 2. Figure 2 and Figure S1 illustrate the processes regulating the wake and sleep phases of the 24 h sleep-wake cycle, *S*(*t*), and the first and second (NREM and REM) phases of sleep cycles, *s*(*t*), during an all-night sleep episode. Figure 3 and Figure S2 show these processes during 12 naps following sleep deprivation and sleep restriction. Particularly, Table 2 demonstrates that the same set of parameters can be used to simulate these two datasets illustrated in Figure 2, Figure 3, Figures S1 and S2.

Table S1 in Supplementary Material contains the preliminary results of simulations of two different spectral EEG indexes of sleep regulation, the traditional index, SWA measured as ln-transformed spectral power densities averaged in the range 1–4 Hz, and PC1 score. When SWA can reflect the contribution of both drives for sleep and wake [27], PC1 score was proposed to be a separate indicator of the drive for sleep, while the opposing drive, for wake, can be represented for the second principal component [17,27].

Figure 2, Figure 3, Figures S1 and S2 illustrate a close similarity between the time courses of PC1 score (Figure 2 and Figure 3) and SWA (Figures S1 and S2). It was shown that the results of simulations of the time courses of one of the indexes (Figure 2 and Figure 3) can be used for fitting the time courses of another index (Figures S1 and S2).

## 5. Conclusions

For four decades, the two-process conceptualization of the process of sleep-wake regulation [1] has become the major contributor to our understanding of the basic mechanisms underlying the sleep-wake cycle. Here, the model of Process S was extended by its combination with a formally identical model of Process s underlying NREM-REM sleep cycles during the sleep phase of the sleep-wake cycle. Several model-based predictions of the *in silico* study were supported in the simulation study of the time courses of spectral EEG indicators of these processes S and s. The presented model provided a parsimonious mathematical description of the complex and varying shape of fluctuations in the indicators of the processes S and s in a sequence of sleep cycles. It was shown that a shape of the sleep cycle can be described as an alternation of simple exponential functions only after the return of the setpoint to its baseline level in the last sleep cycle of an all-night sleep episode. However, an exponential decay of the setpoint governed by Process S causes the deviation of the shapes of previous sleep cycles. It was also predicted and confirmed that the duration of REM sleep gradually increases in a sequence of NREM-REM sleep cycles of an all-night sleep episode. Thus, the results of applying the presented model for prediction and simulation of sleep cyclicities provided an additional, model-based argument in support the hypothesis of the thermoregulatory function of NREM-REM sleep cycles. The results were interpreted as indicating that sleep cyclicities can reflect the work of the brain thermostat that heats or cools to a setpoint brain temperature. By seeking to reduce the error between the setpoint and current temperature, this thermostat produces NREM-REM sleep cycles. It was also demonstrated that, since further brain heating occurs during prolongation of wakefulness, a setpoint of brain temperature requires correction by resetting it to a higher level prior to sleep onset. Such correction reveals itself in the time course of Process S proposed by the classical two-process model of sleep-wake regulation [1]. Such prolongation of wakefulness causes a longer brain heating and leads to the initiation of sleep at a higher setpoint, whereas a nap terminates brain heating earlier, thus causing the initiation of sleep at a lower setpoint. The proposed model of regulating processes underlying sleep cyclicities requires further testing in mammal species with monophasic sleep. If supported, it can provide a valuable framework for understanding the contribution of sleep regulators to the process of thermostatic brain cooling and warming.

## Figures and Tables

**Table 1:** Three processes involved in regulation of the sleep-wake and sleep cycles.

#	Process	*s*(*t*)	*S*(*t*) or *s_u_*(*t*)	*C*(*t*)
1	Formula	(4)	(1); (3,5) after exclusion of (2)	(2) in (1)
2	Name of regulatory mechanism	Ultradian (internal) somnostat	Circadian (external) somnostat	Circadian (clocks) rhythmostat
3	Purpose of regulation	Control of deviations above and below a setpoint	Tuning this setpoint depending upon a previous wake duration	Entrainment and control of period of *S*(*t*)
4	Period of cycle	Approximately 90 min	Approximately 24 h	Entrained to 24 h
5	Phases of cycle	Two: one buildup and one decay	Two: one buildup and either decay or unchanged level	Rising and falling limbs of sinusoid
6	Shape of fluctuations	Inverse exponential function and exponential function	Inverse exponential function and alternation of exponential function with horizontal line	Sine function

**Notes.** The proposed model includes three major terms representing three interacting processes of sleep-wake regulation (Formulas (1)–(5)); #: Comparison of most important characteristics of the sleep-regulating processes. See also Section 4 and Supplementary Material for the descriptions of formulas and parameters of these processes in more detail.

**Table 2:** Parameters of the processes regulating the sleep-wake and sleep cycles.

Process	Parameters		Sleep	Naps
Circadian (external) somnostat, *S*(*t*)	Inverse exponential buildup during wake phase (1a,3a) and exponential decay during some of intervals of sleep phase (1b,3b) of circadian (external) somnostat, *S*(*t*) (1,3)	*S_b_* (lowest allowed decay) at *t1*	0.22	0.23
		*S_d_* (highest allowed buildup) at *t2*	1.10	1.10
		*S_l_* (lower asymptote)	0.20	0.20
		*S_u_* (upper asymptote)	1.41	1.41
		*T_d_* (phase constant for decay), h	1.40	1.40
		*T_b_* (phase constant for buildup), h	12.01	12.01
Circadian (clocks) rhythmostat, *C*(*t*)	24 h sine shape modulation *C*(*t*) (2) of parameters of buildup (1a) and decay phases (1b) of *S*(*t*) (1)	φ_max_ (circadian peak), clock h	0.00	0.00
		*A* (circadian amplitude)	0.00	0.00
		*τ* (entrained circadian period), h	24.00	24.00
		*k* (twofold impact of circadian term)	2.00	2.00
Ultradian (internal) somnostat, *s*(*t*)	Inverse exponential buildup during the first (NREM) phases (4a) and exponential decay during the second (REM) phases (4b) of ultradian (internal) somnostat, *s*(*t*) (4)	*s_b_* (lowest allowed decay)	−0.30	−0.30
		*s_d_* (highest allowed buildup)	0.22	0.22
		*s_l_* (lower asymptote)	−0.34	−0.34
		*s_u_* (upper asymptote) = *S*(*t*) (3)	0.70	0.70
		*t_d_* (phase constant for decay), h	0.36	0.36
		*t_b_* (phase constant for buildup), h	0.22	0.22
	Initial times for buildup (1a,3a) and decay phases (1b,3b) of *S*(*t*)	*t2* (sleep onset), clock h	23.00	23.00
		*t1* (sleep offset), clock h	7.00	6.00

**Notes.** Parameters of the process regulating the sleep and wake phases of the 24 h sleep-wake cycle and the process regulating the first (NREM) and the second (REM) phases of approximately 90 min sleep cycles, i.e., “external” and “internal” somnostats, *S*(*t*) (Formulas (1) and (3)) and *s*(*t*) (Formula (4)), respectively. Sleep and Naps: The simulations suggested that practically identical parameters might be used for fitting data obtained in two—sleep and nap—studies, i.e., during a normal all-night sleep episode and in 12 experimental napping attempts either after total sleep deprivation or after sleep restricted from 23.00 to 6.00. See also Table S1 illustrating that (1) the parameters can be also rather similar in simulations of two different spectral EEG indexes of sleep regulation, PC1 score (score on the 1st principal component of the EEG spectrum) and SWA (slow-wave activity measured as ln-transformed spectral power densities in the range 1–4 Hz), and (2) these parameters did not change much after accounting for the circadian influence, *A* = 0.18 in Formula (2), on *s*(*t*) and *S*(*t*) (Formula (1)). Clock time is given in decimal hours.

## Data Availability

Data of the studies described in Supplementary Material are available on request from the author.

## Supplementary Materials

The following supporting information can be downloaded at: https://www.mdpi.com/article/10.3390/clockssleep6010008/s1. Supplementary Material contains more details on: (1) models of thermostatic, somnostatic and rhythmostatic regulators and (2) calculation of the time courses of the spectral EEG indicators of sleep drive, [28,29,30].
