# Supplementary material for: Can the Brain’s Thermostatic Mechanism Generate Sleep-Wake and NREM-REM Sleep Cycles? A Nested Doll Model of Sleep-Regulating Processes
Source: Clocks Sleep. 2024 Feb 19;6(1):97–113. doi: 10.3390/clockssleep6010008 (PMC10885066; doi:10.3390/clockssleep6010008)
Supplement: Supplementary file 1 [file clockssleep-06-00008-s001.zip › clockssleep-2740895-supplementary.pdf]

Supplementary Material

to:

Can the Brain's Thermostatic Mechanism Generate Sleep–Wake and NREM–REM Sleep Cycles? A Nested Doll Model of Sleep-Regulating Processes

S1. Models of Thermostatic, Somnostatic and Rhythmostatic Regulators

This section contains a brief description of the relevance of the models of somnostats and rhythmostats to model the technical counterparts of sleep regulators, the model of a relay thermostat [2]. Formal identity of regulatory processes was noted by the authors of the classical model of the homeostatic process of sleep–wake regulation [1] who coined the term "somnostat" to emphasize that "the system acts like a thermostat that switches off at a higher threshold than it switches on" [1]. In other words, hysteresis is the basic feature of such regulating mechanisms because, like the thermostatic regulator, the hypothetical sleep regulator named "somnostat" or "homeostatic process" or "Process S" produces hysteresis. Therefore, it was further proposed [3] that a model of a relay thermostat [2] can be applied for modeling Process S [1].

S1.1. A Model of Relay Thermostat

The model of this device was previously described in [2-4]. If, in this thermostat model, *S(t)* is a current level of temperature, the equation for S can be obtained by considering the energy balance in a thermostatic system:

$$\dot{E} = \dot{E}_D + \dot{E}_A \quad \text{(S1)}$$

where $\dot{E}$ is the production of energy by a heating device, and $\dot{E}_D$ and $\dot{E}_A$ are dissipated and accumulated energy, respectively.

When the heater is switched either on or off, $\dot{E}$ remains constant, and the dissipation of heat is proportional to temperature, S:

$$\dot{E}_D = k * S \quad \text{(S2)}$$

If thermal capacity m is also a constant, the accumulation of energy is proportional to the thermal capacity:

$$\dot{E}_A = m * \dot{S} \qquad \text{(S3)}$$

After introducing (2) and (3) in (1):

$$\dot{E} = m * \dot{S} + k * S, \qquad \text{(S4)}$$

and the time constant and the limits for temperature can be defined as:

$$\dot{E}/_k = \begin{cases} S_u \\ S_l \end{cases}$$

where S_u and S_l are the maximal and minimal temperature, respectively. After their introducing, the equation (4) becomes:

$$T * \dot{S} + S \qquad \text{(S5a)},$$

$$= \begin{cases} S_u \text{ for buildup phase, i.e., when the heater is switched on} \\ S_l \text{ for decay phase, i.e., when the heater is switched off} \end{cases} \qquad \text{(S5b)}$$

For the initial t=0, S=S_b and S=S_d, the equations (5) have the following solutions:

$$S=S_u-(S_u-S_b)*e^{(-t/T)} \text{ for buildup phase} \qquad \text{(S6a)},$$

$$S=S_l-(S_d-S_l)*e^{(-t/T)} \text{ for decay phase} \qquad \text{(S6b)}$$

If S_S is a temperature setpoint, current temperature $S(t)$ oscillates up and down relative to this setpoint. A delay is expected between temperature measurement and the following switching response. Particularly, it takes time, TT, for a signal to switch the heating device to reach the relay from an analyzer of temperature. Further, a zone of insensitivity is additionally expected. Within this zone, the regulator does not react on deviation of $S(t)$ from S_S, i.e., this is the so-called hysteresis mentioned in [1] in the statement about similarity between a thermostat and a "somnostat", i.e., the homeostatic process or Process S of the classical two-process model. Hysteresis can be defined as the expected deviation of temperature from the target temperature, i.e., the temperature setpoint. This deviation allows for a certain amount of temperature fluctuation above and below the temperature setpoint before heating is

triggered. As the lowest temperature that is allowed below/above the temperature setpoint is reached, heating is triggered to bring temperature closer to the setpoint temperature.

Therefore, the phase transitions (i.e., turning the heater on or off) occur at S=S_b and S=S_d rather than at S=S_S, i.e., at the temperature setpoint. The values of S can be calculated for five time points, the beginning of temperature buildup, t_b, the moment when temperature reaches the setpoint after the beginning of buildup, t_{bs}, the beginning of temperature decay, t_d, the moment when temperature reaches the setpoint after the beginning of decay, t_{ds}, and the moment when the cycle starts again, t_{b+T}:

$$= S_s,\ \mathrm{S}(t_d) = S(t_{bs} + TT) = S_d \text{ for buildup phase} \quad \text{(S7a)},$$

$$= S_s,\ S(t_{b+\tau}) = S(t_{ds} + TT) = S_b \text{ for decay phase} \quad \text{(S7b)}$$

The following equations are obtained after including these equations in (S6) and excluding t_{bs} and t_{ds}:

$$S_d = S_u - (S_u - S_s) * e^{(-TT/T)} \quad \text{(S8a)},$$

$$S_b = S_l - (S_s - S_l) * e^{(-TT/T)} \quad \text{(S8b)},$$

$$G = \frac{1}{2}(S_d - S_b) = \frac{1}{2}(S_u - S_l) * [1 - e^{(-TT/T)}] \quad \text{(S9)},$$

where G is an amplitude of thermostatic oscillations.

The following are the limits for the equation (9):

When $TT \rightarrow 0$, $G \rightarrow 0$, and when $TT \rightarrow \infty$, $G \rightarrow \frac{1}{2}(S_u - S_l)$.

S1.2. A Model of Somnostatic Regulation of Sleep and Wakefulness

In order to achieve the full correspondence between a thermostat (8) and the model of a "somnostat" (i.e., the homeostatic process proposed in [1]), it is necessary to additionally suggest the difference in T (time constant) for the buildup and decay phases of $S(t)$:

$$T_b = Waking\ time / \ln\,[(S_u - S_b)/(S_u - S_d)] \quad \text{(S10a)},$$

$$T_d = (24 - Waking\ time)\ /\ \ln\,[(S_d - S_l)/(S_b - S_l)] \quad \text{(S10b)}$$

where T_b and T_d are the time constants for the phases of buildup and decay (wakefulness and sleep, respectively).

S1.3. A Model of Rhythmostatic Regulation of Sleep and Wakefulness

The rhythmostatic version of the two-process model [3] postulates that rhythmostasis of the body rhythms [22], including such rhythms as the sleep–wake cycle, can be simply achieved via the modulating the influence of the circadian clocks on all parameters of the homeostatic process of the classical version of the two-process model [1]. Such influence on the homeostatic (somnostatic) process, $S(t)$ [1], is incorporated in the model by adding the simplest periodic (sine) function with a circadian period, $C(t)$, to each parameter of Process S [3].

When the initial times for the buildup (wake) and decay (sleep) phases of the sleep–wake cycle are *t1* and *t2* (e.g., rise- and bedtime, respectively), the time course of the sleep–wake regulatory process $S(t)$ can be described as:

$$S(t) = [S_u + C(t)] - \{[S_u + C(t)] - S_b\} * e^{-\frac{t-t1}{[Tb - k * C(t)]}} \quad \text{(S11a)},$$

$$S(t) = [S_l + C(t)] - \{S_d - [S_l + C(t)]\} * e^{-\frac{t-t2}{[Td - k * C(t)]}} \quad \text{(S11b)},$$

where

$$[illegible](2\pi * t/\tau + \varphi_0) \quad \text{(S12)}$$

is a sine function with a circadian period τ. It represents the modulation influence of the circadian clocks on $S(t)$. In the calculations of alternations between wake and sleep phases, this period is assigned to 24 h, i.e., the circadian clocks are suggested to remain in sync with (are entrained to) the light–dark cycle with, on average, the 24 h period.

S1.4. Contribution of the Circadian Clocks to the Regulation of the Sleep–Wake Cycle

Since the circadian sleep–wake cycle and NREM–REM sleep cycle have different time scales, it is assumed that, in the present simulations, this circadian modulation of the regulated parameter within each approximately 90 min sleep cycle cannot strongly influence the time course of this parameter (see also 2.4 of this Supplementary Material). However, it is important to emphasize that circadian clocks are absolutely necessary for keeping the sleep–wake cycle in sync with the external 24 h cycle of light and darkness.

The model [3,4] represents the somnostatic (homeostatic) process, *S(t)*, in the traditional way as the alternations of an inverse exponential curve for the wake phase (1a) and an

exponential curve for the sleep phase (1b) [1]. However, the specific feature of the rhythmostat model [3] is the additional postulation of the modulation of parameters of *S(t)* by body clocks. It is represented by a 24 h sine-form function *C(t)* (2) in (1) [3]. For instance, when sleep duration and timing are determined endogenously, *S(t)* oscillates between its highest and lowest buildup and decay, $S_d(t)$ and $S_b(t)$, that are the habitual sleep onset and offset. However, humans do not always obey the signal of falling asleep sent by this internal regulator. They might be forced to (or eager to voluntarily) delay the time to go to bed. Therefore, their wake phase might be extended due to prolongation of wakefulness. Moreover, they can reduce their sleep phase by early wake-ups on weekdays, i.e., before the time of spontaneous wakings-up at $S_b(t)$.

During such a prolongation of wakefulness, *S(t)* continues its building-up after crossing $S_d(t)$, but, despite this additional buildup, the following sleep episode is always terminated at endogenously determined $S_b(t)$. Due to early wake-ups, *S(t)* does not reach $S_b(t)$, but, despite this early termination of sleep, the following sleep episode is always initiated at $S_b(t)$. The rhythmostatic influence on the parameters of the sleep–wake regulatory process ensures the invariantly rapid return to the baseline sleep timing after the first night of ad lib sleep [4].

Thus, the major adaptive significance of the control of the circadian clocks over the sleep–wake regulatory process formalized by adding *C(t)* in the equations for *S(t)* is in the possibility of reestablishment of the times of sleep onset, sleep offset and sleep duration determined by this endogenous sleep–wake regulatory process on the short interval of one night of ad lib sleep following any shifts of the times of going to bed and/or getting up [4]. Therefore, this mechanism is named a “rhythmostat” in [3] (see [4] for more details and illustrations).

S1.5. Comments on the Regulation of the Sleep–Wake and Sleep Cycles in Humans

The regulator generating NREM–REM sleep cycles from which the sleep phase of the human sleep–wake cycle consists is the low-tier regulator (Process s) and the higher-tier regulator (Process S) tunes the setpoint of Process s. Namely, the purpose of this high-tier regulator is to tune up the low-tier regulator to adjust its setpoint to a duration of a preceding wake phase of the sleep–wake cycle. The longer the duration of the preceding wake phase of Process S, the higher the level of the setpoint of Process s in the beginning of the following sleep phase of the sleep–wake cycle and the steeper the decay of this setpoint of Process s during NREM phases in the first three sleep cycles of an all-night sleep episode.

Given that the work of the high-tier regulator is usually documented by measuring mean levels of slow-wave activity (SWA) in the first three sleep cycles (e.g., [1]), it is also expected to document the work of the low-tier regulator by measuring SWA, but across shorter time intervals, within each of sleep cycles constituting an all-night sleep episode. If the decay of the mean level of SWA from one sleep cycle to another can serve as an indicator of the decay of the setpoint of Process s, a buildup and the following decay of SWA within each of these cycles can serve as an indicator of the oscillating process generated by Process s. The buildup of SWA in the first sleep cycle is expected to be steeper after a longer interval of preceding wakefulness than that after a shorter interval of preceding wakefulness because sleep after a longer interval is initiated at a higher setpoint than sleep initiated after a shorter interval.

The well-established observation of the decay of SWA from one sleep cycle to another is interpreted in [1] as indicating that, within each of the first three sleep cycles, an inverse exponential buildup of SWA in the beginning and middle of each of these cycles is distorted by an exponential decay of the setpoint of process S generating approximately 90 min alternations between NREM and REM sleep. The approximately 24 h sleep–wake homeostatic process of the classical two-process model [1] (Process S) is designed for governing such a decay of the setpoint of the regulator of sleep cycles (Process s). In other words, the model postulates that one of the processes, Process S, is required for the determination of the setpoint of another process, Process s, during the sleep phase of the approximately 24 h sleep–wake cycle. After a longer wake phase of this cycle (more than two-thirds of the cycle length), the setpoint is elevated at a rather high level, and, therefore, it decays faster during the following sleep phase, while, after a shorter wake phase of this cycle (less than two-thirds of the cycle length), the setpoint is elevated at a lower level and, therefore, it decays slower throughout the following sleep phase (Figure 1C).

S2. Calculation of the Time Courses of the Spectral EEG Indicators of Sleep Drive

This section contains a brief description of data used for the present simulations and some results of simulations that were not included in Table 2 and Figures 2 and 3. Simulations of the processes of homeostatic regulation of the sleep–wake and sleep cycles were performed through fitting the time courses of their electroencephalographic (EEG) indicators of sleep regulation [1,26] obtained from the EEG recordings collected in two studies. The methods,

results and illustrations of these two studies were described in previously published papers [27-30].

The experiments were performed in accordance with the ethical standards laid down in the Declaration of Helsinki. The experimental protocols were approved by the Biomedical Research Ethics Committee of the Siberian Branch of the Russian Academy of Medical Sciences, SB RAMS (No. 18 from 12th from October, 2008, Institute of Physiology of SB RAMS). Informed written consent was obtained from each study participant.

S2.1. Study Participants and Study Protocol

The dataset of the first study includes the spectral EEG indexes calculated from the polysomnographic recordings obtained from 18 male cadets of the Novosibirsk Military High School. Ages ranged from 18 to 22 years. Sleep of the study participants prior to the experiments was chronically restricted because their everyday times for going to bed and waking up were 23:30 h and 06:30 h, respectively. The experiments were carried out in the laboratory complex of the institute on nine weekends, between Saturday morning and Monday morning, with two participants being studied each weekend [29].

Both participants were kept continuously awake until 23:00 h. Then, one of them (sleep-restricted) was allowed to sleep in the sleep laboratory until 06:00 h. The other (sleep-deprived) was kept awake for the whole night. The next 24 hours were scheduled for multiple naps. The applied ultradian sleep–wake paradigm consisted of 100 min of wakefulness followed by a 20 min napping attempt. The first sleep attempt was scheduled at 6 a.m. and 7 a.m. for the sleep-deprived and the sleep-restricted participant, respectively (see the X axis in Figure 3B and Figure S2B). A participant was asked to sleep lying in bed in a sound-attenuated and completely darkened room of the sleep laboratory for 20 min with closed eyes. Then, he was taken out of the sleep laboratory to stay in other rooms of the laboratory complex together with the study personnel for the entire 100 min time interval between consecutive sleep attempts.

To prevent unintended sleep, the participants were constantly engaged in research activities and social interactions. Throughout each 100 min time interval of wakefulness, the same set of parameters were measured (basic heart and respiratory rates, heart cost of 3 min physical load on bicycle ergometer, axillary temperature, subjective time estimation,

subjective vigilance score, etc.). Moreover, performance of the study participants was additionally assessed using a psychomotor test. During the breaks between such measurements, the participants completed chronobiological and psychological questionnaires. If they did not participate in such research procedures, they were engaged in talking, reading, watching TV, playing computer games, etc. They were asked to avoid vigorous physical activity, exposure to light brighter than 500 lux, smoking or taking any medications or any alcohol and caffeinated beverages. During night hours, only light snacks and soft drinks were offered, and, during daytime hours, the participants visited the canteen in the same building to have breakfast (between 8:30 and 9:00), lunch (between 13:30 and 14:00) and dinner (between 18:30 and 19:00). Rectal temperature and heart rate were recorded automatically every min, and urine samples were collected for further measurement of levels of epinephrine and norepinephrine [29].

The dataset of the second experimental study includes the spectral EEG indexes computed from the recordings of baseline sleep episodes in 14 female participants. Ages ranged from 17 to 55 years old (average ± SD: 35.4 ± 11.0 yrs). Each participant was studied individually in two identical experiments, and each experiment started with admission to the sleep laboratory for an adaptation night. The next night, polysomnography started after lights-off at 23:00 h, and sleep was allowed until complete awakening [29].

S2.2. The EEG Recording and Analysis

The EEG records were obtained using a standard monitoring montage that included one chin electromyogram channel, two electrooculogram channels and the remaining EEG channels. The reported data were taken from the Cz-A1 derivation of the International Ten-Twenty System of Electrode Placement (i.e., vertex of the head vs. left mastoid). To fix the electrodes, Ten20 conductive paste was used (Nicolet Biomedical, Madison, Wisconsin, USA). Polysomnographic data were collected via an eight-channel Medicor polygraph (EEG8S, Micromed, Hungary). Two independent judges visually scored sleep stages in the polysomnographic records. Epochs with discrepant scores were reexamined by both judges together to produce consensus scores. Each epoch was categorized according to the conventional criteria as wakefulness, stages 1, 2 and 3 of NREM sleep, REM sleep or movement.

The polysomnographic signals were high-band-pass (0.5 Hz), low-band-pass (64 Hz) and notch-filtered (50 Hz), digitized at a sampling frequency of 128 Hz and stored on a hard disc. The artifacts were detected on 5 sec intervals, and absolute power values (μV^2) were obtained for the artifact-free 5 sec intervals of the EEG signal using a fast Fourier transform algorithm. The spectra were reduced to single-Hz bin widths by calculating the mean absolute powers over adjacent frequencies and then further reduced by averaging the artifact-free spectra within each one-minute interval.

The SPSS statistical software package was used for all statistical analyses [27-30].

S2.3. Spectral EEG Indexes of Sleep-Regulating Processes

One-minute absolute single Hz powers in the frequency range from 1 to 16 Hz were converted into a natural logarithmic (ln) scale and subjected to principal component analysis. In order to calculate scores on the first (largest) principal component (PC1), these 16 values were optimally weighted in accord with their loadings on this component and then summed. The methodology of principal component analysis of the EEG spectrum, the pattern of loadings of first principal component, and the time courses of spectral powers and scores on the principal components during all-night sleep and within 20 min intervals of multiple naps were reported in more detail earlier [27-30]. The time courses of PC1 score are illustrated in Figures 2 and 3.

Moreover, the same ln-transformed powers were averaged over four 4 Hz intervals roughly corresponding to the conventional delta, theta, alpha and sigma bands of the EEG spectrum (i.e., 1-4 Hz, 5-8 Hz, 9-12 Hz and 13-16 Hz, respectively). The time courses of the ln-transformed delta powers (i.e., 1-4Hz) or, in other terms, SWA, are illustrated in Figures S1 and S2. SWA is conventionally used to quantify the number and amplitude of slow waves through a spectral analysis of the EEG signal. It serves as an index of the presence and depth of NREM sleep [26] and as an index of the homeostatic process of sleep–wake regulation [1]. The effect of duration of wakefulness on SWA was well established and the time course of SWA became the traditional spectral EEG indicator of the parameter regulated by Process S (see the main text).

Data of the time courses of the spectral EEG indexes from the first and second studies were used for simulating the time courses of hypothetical regulatory processes in an all-night

(baseline) sleep episode (Figures 2 and S1) and during multiple 20 min napping attempts (Figures 3 and S2). Each of the figures in the main body of the article shows the time courses and simulations of PC1 score (Figures 2 and 3). The time courses of SWA were found to be very similar to the time courses of PC1 score (Figures S1 and S2). Table S1 illustrates the similarity of the model's parameters for two indexes revealed in preliminary simulations that was suggested by the applying the least-squares method (see SS at the bottom of Table S1).

S2.4. Some Results of Preliminary Simulations

The preliminary simulations of time courses of spectral EEG indicators of sleep regulation were performed using the least-squares method. Comparison of simulations on the sum of squares (SS) provided results allowing the simplification of the model by excluding the circadian term (2). Since the human circadian sleep–wake cycle and NREM–REM sleep cycle have different time scales, it can be expected that the circadian modulation (2) of the processes S and s within a short interval of each sleep cycle can be neglected in simulations of such cycles. Preliminary simulations with A=0.18 confirmed this expectation (Table S1).

Table S1. Parameters of the preliminary simulations of *s(t)* in the first four sleep cycles of all-night sleep.

EEG index	PC1 score		SWA	
τ	24.00	24.00	24.00	24.00
φ(rad)	0.00	-0.48	0.00	-0.48
A	0.00	0.18	0.00	0.18
s_{u1}	1.53	0.94	0.86	0.82
s_{u2}	0.92	0.73	0.67	0.61
s_{u3}	0.67	0.61	0.56	0.49
s_{u4}	0.48	0.46	0.42	0.37
s_{d1}	0.86	0.80	0.62	0.67
s_{d2}	0.59	0.62	0.49	0.50
s_{d3}	0.43	0.44	0.38	0.37
s_{d4}	0.32	0.25	0.24	0.23
s_{b1}	-0.34	-0.34	-0.34	-0.34
s_{b2}	0.10	0.10	0.07	0.06
s_{b3}	-0.06	-0.05	-0.06	-0.03
s_{b4}	-0.09	-0.10	-0.09	-0.06
s_l	-0.26	-0.31	-0.21	-0.24
t_b	0.30	0.30	0.30	0.30
t_d	0.44	0.50	0.44	0.50
SS	0.23	0.18	0.14	0.12

Notes: s_{b1}, s_{b2}, s_{b3}, s_{b4}, s_{d1}, s_{d2}, s_{d3} and s_{d4}: Simulated initial values of the EEG indexes for buildup and decay phases of the first four sleep cycles of all-night sleep; and s_{u1}, s_{u2}, s_{u3}, s_{u4}: Simulated value of upper asymptotes in the beginning of these cycles. SS: sum of squares.

Comparison of SS obtained for the simulations with two values of the amplitude of the circadian rhythm (A=0.00 and A=0.18) suggetsted that the results of fitting data are only slightly improved after accounting for the influence of body clocks, $C(t)$, on the parameters of $s(t)$.

Moreover, these preliminary simulations suggested a very close similarity of parameters of the model for fitting time courses of two spectral EEG indicators of sleep regulation, PC1 score and SWA (Table S1).

In particular, this similarity can be seen by comparison of the fit between simulation of the time course of PC1 score and the time course of SWA (Figure 2B vs. S1B, and 3B vs. S2B). Therefore, the results on SWA are illustrated in this Supplementary Material (Figures S1 and S2).

S2.5. The Time Courses of Two Spectral EEG Indexes Illustrated in Figures 2 and 3

The time courses of PC1 score shown in Figure 2 were taken from Figure 4 of one of the previous publications of this dataset [27]. Before averaging data on two all-night sleep episodes in 14 study participants, the boundaries between the first four ultradian sleep cycles were determined. The upward and downward mean-crossing criteria were applied to the time courses of single-minute scores on the first principal component of the EEG spectrum (PC1). The procedure of averaging single-minute indexes of PC1 score within these cycles was performed across an equal number of time intervals of different length (i.e., in order to obtain, in total, values for 38 intervals of each of the analyzed records). The length of each interval of averaging was calculated from the ratio between a period of an analyzed cycle and the mean period of this cycle. Therefore, the lengths of cycles varied from 5 to 16 min around the 9 min length of the interval. Such averaging was performed over two records of each of the 14 participants of the study and over these 14 participants (see also [27] for more details).

The cycle-averaged values shown in Figure 2 were obtained by calculating mean values from a set of individual values obtained for 9 min intervals of each of four cycles. An additional mean value for the final interval of all-night sleep shown in Figure 2A was obtained from data on the two-hour time course preceding complete awakening. This final

value is earlier illustrated in Figure 2 of one of the previous publications that also contains more details on the method of its calculation [29].

The time courses of PC1 score shown in Figure 3 illustrate the buildup of a spectral EEG indicator of sleep regulation in the beginning of a nap initiated after different durations of preceding wakefulness during three-minute intervals of sleep episodes. The first value was calculated by averaging over single-minute values from the last minute of wakefulness to the last minute of stage 1 sleep. The following four values were calculated by simple averaging within the following 3 min intervals of NREM sleep (Figure 3B). The values for each napping attempt were obtained by averaging over five such values (Figure 3A). The standard errors for individual values were calculated by averaging within the groups of nine sleep-restricted and nine sleep-deprived participants of the first study (Figure 3).

The time course values of the indicator of sleep regulation shown in Figure 3A were simulated for the mean interval of sleep obtained for each napping attempt by simple averaging over study participants.

Figures 2 and S1 were designed to illustrate the model-based prediction that, although the experimental or simulated curve does not resemble exponential buildup, they can be explained by interaction between simple exponential curves. The regulator of sleep–wake cyclicity, $S(t)$, changes a setpoint of the regulator of sleep cyclicity, $s(t)$. This setpoint decays exponentially during the first of two phases of sleep cycle during the interval of inverse exponential buildup of the regulated parameter determined by $s(t)$. Due to this decay, the curve consists of the rising and saturation parts on this interval and the amplitude and waveform of sleep cycle varies from one cycle to another.

Figures 3 and S2 were designed to illustrate the prediction that an inverse exponential buildup of the regulated parameter can be either shorter or longer, and, consequently, $S(t)$ tunes the level of the setpoint of the regulator of sleep cycle, $s(t)$, in such a way that this setpoint reaches either the higher or lower level, respectively, in the beginning of the first phase of the first sleep cycle. Therefore, the buildup of the spectral EEG indicators of sleep regulation in the beginning of the first napping attempt is predicted to be different in the condition of the preceding sleep deprivation compared to the condition of preceding sleep restriction. This difference is predicted to disappear in the last napping attempts due to further restriction of sleep (i.e., not longer than 20 min per 2 h interval of the day and night).

S2.6. The Time Courses of the Classical Spectral EEG Index Illustrated in Figures S1, and S2

In Figures S1 and S2, the time courses of SWA were plotted against simulations of PC1 score to illustrate similarity of results obtained for two different spectral EEG markers of sleep regulation.

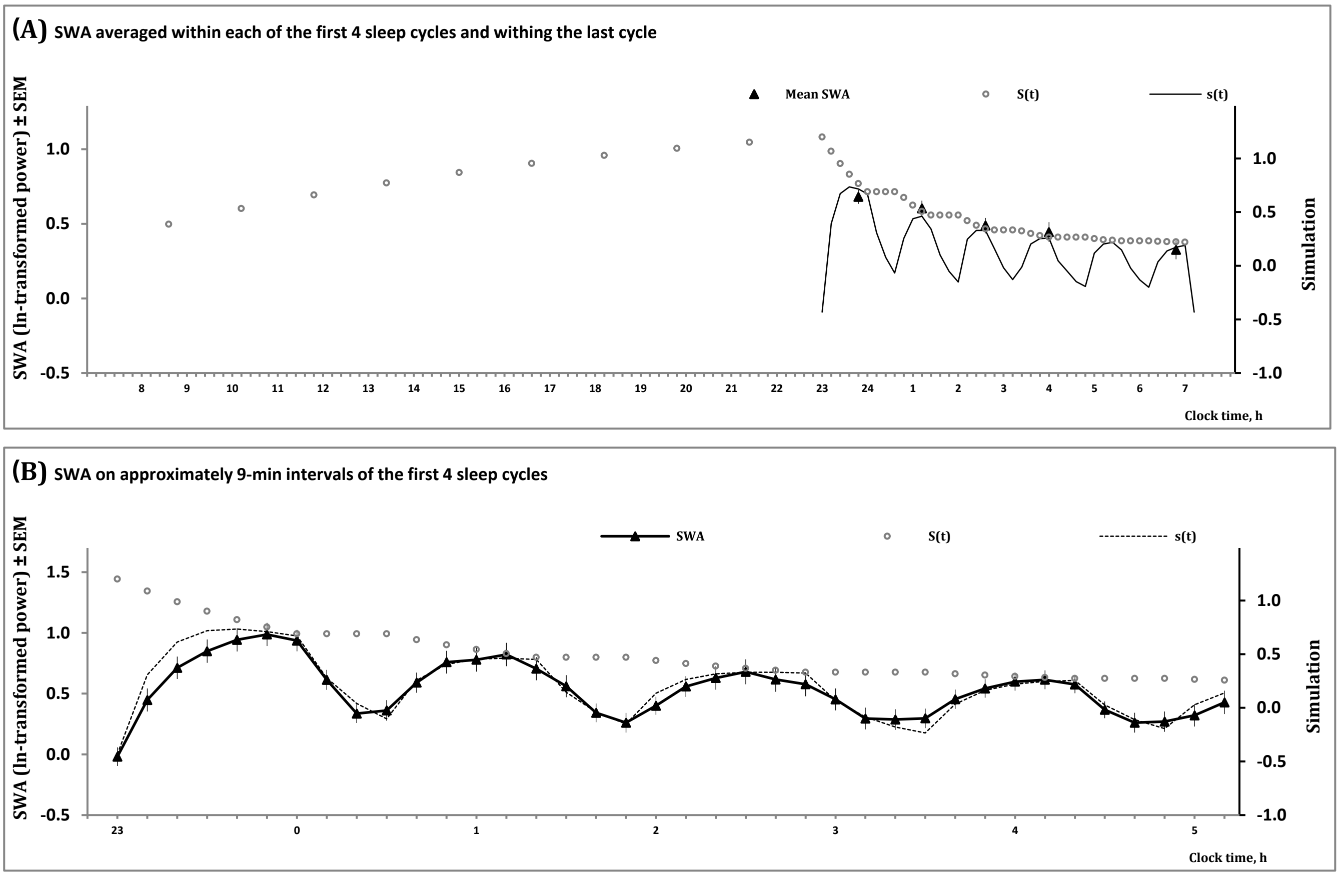

Figure S1. Simulation of sleep homeostasis during all-night sleep and during the first four sleep cycles.

All-night sleep: (A) the process of sleep–wake regulation, *S(t)*, during its two (wake and sleep) phases and the process of NREM–REM sleep regulation, *s(t)*, during sleep; (B) the same processes during the first four sleep cycles. Across each of the intervals of inverse exponential buildup of SWA during the first phase of sleep cycles (4a), Process S governs an exponential decay of the setpoint of Process s (5a). Therefore, a rather complex waveform of the first sleep cycles can be explained by the modulating influence of a mathematically simple exponential decay of the setpoint (5a) on a similarly simple inverse exponential buildup of PC1 score during the first phase of these cycles (4a). See the model's parameters in Tables 2 and S1 and the graphs for PC1 score in Figure 2.

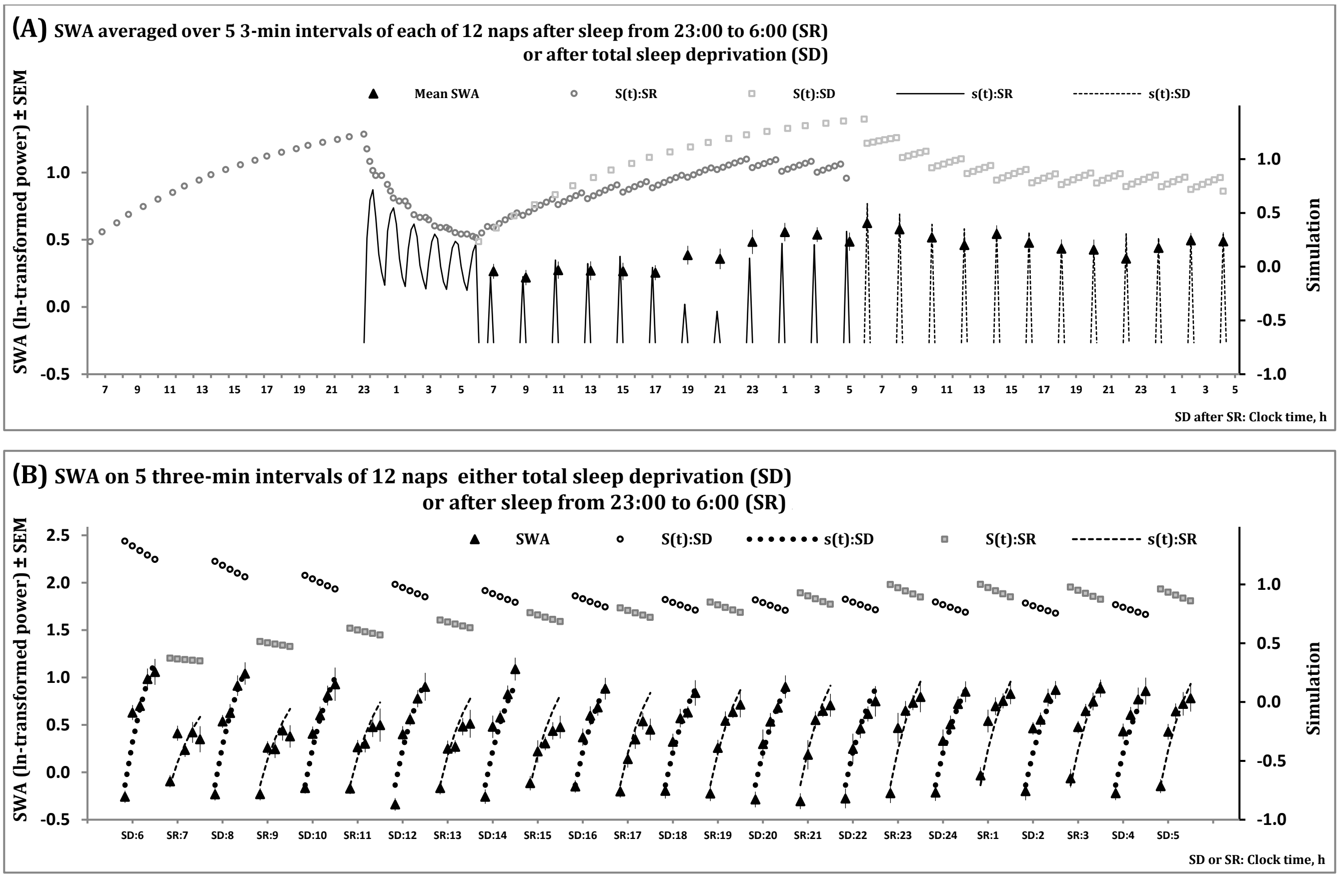

Figure S2. Simulation of sleep homeostasis in 12 naps after sleep deprivation and restricted sleep.

Twelve napping attempts during a 24 h time interval either after restriction of sleep (SR) or after total sleep deprivation (SD): (A) the process of sleep–wake regulation, *S(t)*, during two 24 h time intervals and the process of NREM–REM sleep regulation, *s(t)*, during restricted sleep and 12 napping attempts. *S(t)* is shown on the left for two (wake and sleep) phases of the process of sleep–wake regulation in the condition of sleep restriction and it is shown in the center for total sleep deprivation. The following 12 napping attempts during the 24 h time interval are shown in the center and on the right, respectively; (B) the same processes are shown during the first 12 min of sleep episodes in each of 12 napping attempts after either total sleep deprivation or sleep restricted to 7 hours. Naps were set either at every even hour starting at 6 a.m. or at every odd hour starting at 7 a.m. The curves of S(t):SD and S(t):SR on the intervals of each nap illustrate the effect of preceding wakefulness on the level of the setpoint determining the steepness of buildup. Since the SD group did not sleep while the SR group slept for 7 hours prior to napping attempts, the buildup of SWA after initiation of sleep in the first napping attempts was profoundly steeper in the former than in the latter group. See the model’s parameters in Tables 2 and S1 and the graphs for PC1 score in Figure 3.

References

1. Daan, S.; Beersma, D.G.M; Borbély, A.A. Timing of human sleep: recovery process gated by a circadian pacemaker. *Am J Physiol Regul Integr Comp Physiol.* **1984**, *246*, R161-R178.

2. Magnus, K. *Schwingungen: Eine Einführung in die theoretische Behandlung von Schwingungsproblemen*. BG Teubner Verlagsgesellschaft, Stuttgart. **1976**.

3. Putilov, A.A. The timing of sleep modelling: circadian modulation of the homeostatic process. *Biol. Rhythm Res.* **1995**, *26*, 1-19.

4. Putilov, A.A. Reaction of the endogenous regulatory mechanisms to early weekday wake-ups: a review of its popular explanations in the light of model-based simulations. *Front. Netw. Physiol.* **2023**, *3*, 1285658.

22. Strughold, H. Rhythmostasis - a fundamental life characteristic aerospace medical aspect. *Riv. Med. Aeronaut. Spaz.* **1971**, *34*, 168–175.

26. Greene RW, Frank MG. Slow wave activity during sleep: functional and therapeutic implications. *The Neuroscientist* **2010**, *16*, 618–633.

27. Putilov, A.A. Principal components of electroencephalographic spectrum as markers of opponent processes underlying ultradian sleep cycles. *Chronobiol Int.* **2011**, *28*, 287-299.

28. Putilov, A.A. Principal component structure of wake-sleep transition: quantitative description in multiple sleep latency tests. *Somnologie.* **2010**, *14*, 234-243.

29. Putilov, A.A. Prospects of using electroencephalographic signatures of the chronoregulatory processes for meaningful, parsimonious and quantitative description of the sleep-wake sub-states. *Biol Rhythm Res.* **2011**, *42*, 181-207.

30. Putilov, A.A. The EEG indicators of the dynamic properties of sleep-wake regulating processes: comparison of the changes occurring across wake-sleep transition with the effects of prolonged wakefulness. *Biol Rhythm Res* **2013**, *44*, 621–643.

Note. References [1-4, 22, 26–30] are cited in the main text and are included in the reference list of the main text under the same numbers.
